# Theoretical Model to Ground Research on Medicaid Cost Savings From Elder Mistreatment Intervention

**DOI:** 10.1093/geroni/igaf122.1426

**Published:** 2025-12-31

**Authors:** Tony Rosen, Elaine Gottesman, Alyssa Elman, Yongkang Zhang, Yiyuan Wu, Jiani Yu, Zachary Gassoumis, Kenneth Steinman

**Affiliations:** Weill Cornell Medicine / NewYork-Presbyterian Hospital, New York, New York, United States; New York-Presbyterian Hospital, New York, New York, United States; Weill Cornell Medicine, New York, New York, United States; Weill Cornell Medicine, New York, New York, United States; Weill Cornell Medicine, New York, New York, United States; Weill Cornell Medicine, New York, New York, United States; UTHealth Houston, Houston, Texas, United States; The Ohio State University, Columbus, Ohio, United States

## Abstract

Measuring the costs associated with elder mistreatment (EM) and identifying potential downstream cost savings from intervention and prevention efforts are essential research goals to influence policy. Given that Medicaid has a central role in funding healthcare for poor and institutionalized older adults, we hypothesized that EM and response may have a substantial impact on associated costs. We developed a theoretical model describing in detail the multiple pathways by which EM may lead to additional Medicaid expenditures, including through: (1) unplanned high-intensity, high-cost acute health care, (2) inability set up/maintain adequate home and community-based services, (3) financial exploitation diverting and older adult’s income and draining assets, (4) long-term care (LTC) placement, and (5) spend down of private assets after LTC admission. We used the model to identify 8 discrete targets for intervention. This model may guide future research examining the impact of EM on total per capita Medicaid cost and whether EM increases the likelihood of: qualifying for Medicaid, conversion from community to institutional Medicaid, and/or spending down from private payment in an LTC to Medicaid. Also, the impact of intervention and prevention efforts at specific targets may be measured. This theoretical model also informs research strategies, which may include close examination of natural experiments, longitudinal examination of a cohort of older adults experiencing EM before and after identification/intervention, and comparative studies using control groups.

